# The Rapidly Evolving Scenario of Acoustic Voice Analysis in Otolaryngology

**DOI:** 10.7759/cureus.73491

**Published:** 2024-11-11

**Authors:** Marco Fantini, Gabriele Ciravegna, Alkis Koudounas, Tania Cerquitelli, Elena Baralis, Giovanni Succo, Erika Crosetti

**Affiliations:** 1 Ear, Nose, and Throat Unit, Koelliker Hospital, Turin, ITA; 2 Ear, Nose, and Throat Unit, San Feliciano Hospital, Rome, Italy; 3 DataBase and Data Mining Group (DBDMG) Department of Control and Computer Engineering (DAUIN), Polytechnic University of Turin, Turin, ITA; 4 Ear, Nose, and Throat Clinic, Head and Neck Cancer Unit, San Giovanni Bosco Hospital, Turin, ITA; 5 Department of Oncology, University of Turin, Turin, ITA

**Keywords:** acoustic analysis, acoustic voice quality index, artificial intelligence, vocal quality, voice

## Abstract

The field of voice analysis has experienced significant transformations, evolving from basic perceptual assessments to the incorporation of advanced digital signal processing and computational tools. This progression has facilitated a deeper understanding of the complex dynamics of vocal function, particularly through the use of acoustic voice analysis within a multidimensional evaluation framework. Traditionally, voice analysis relied on parameters such as fundamental frequency, jitter, shimmer, and noise-to-harmonic ratio, which, despite their utility, have faced criticism for variability and lack of robustness. Recent developments have led to a shift toward more reliable metrics such as cepstral measures, which offer improved accuracy in voice quality assessments. Furthermore, the integration of multiparametric constructs underscores a comprehensive approach to evaluating vocal quality, blending sustained vowels, and continuous speech analyses. Current trends in clinical practice increasingly favor these advanced measures over traditional parameters due to their greater reliability and clinical utility. Additionally, the emergence of artificial intelligence (AI), particularly deep learning, holds promise for revolutionizing voice analysis by enhancing diagnostic precision and enabling efficient, non-invasive screening methods. This shift toward AI-driven approaches signifies a potential paradigm change in voice health, suggesting a future where AI not only aids in diagnosis but also the early detection and treatment of voice-related pathologies.

## Introduction and background

The field of voice analysis has undergone a profound evolution over the last century, transitioning from initial perceptual evaluations to the integration of complex digital signal processing techniques [1-3]. This advancement underscores a pivotal shift in the methodologies employed in phonetics, speech science, and laryngology, aimed at elucidating the complex dynamics of vocal fold vibrations and the acoustic characteristics imparted by the vocal tract’s resonance [4-6]. The advent of sophisticated computational tools and software has significantly enhanced the precision and depth of these analyses, supporting a comprehensive understanding of voice quality [7,8].

Acoustic voice analysis is part of a broader, multidimensional approach to voice evaluation, encompassing perceptual voice assessments, self-evaluations, laryngostroboscopic examinations, and acoustic measurements. This holistic framework is essential for a thorough investigation of vocal function, offering insights into the nuanced interplay between physiological mechanisms and acoustic output [9,10].

The clinical utility of acoustic analysis extends beyond mere documentation, serving as a critical component in monitoring treatment progress and facilitating evidence-based therapeutic decisions. By providing objective, quantifiable metrics, it complements perceptual evaluations and laryngostroboscopic findings, enriching the diagnostic process and enhancing the objectivity of treatment outcomes assessment [7,9]. In the research domain, the exploration of acoustic properties has contributed significantly to advancing our knowledge of vocal physiology and the effects of various conditions on voice production [11]. This research has catalyzed the development of innovative therapeutic interventions and preventive strategies, contributing to the optimization of vocal health and performance. In summary, traditional acoustic voice analysis, within the context of a multidimensional evaluation model, embodies the convergence of science and clinical practice in voice care.

## Review

Limitations of traditional acoustic parameters for voice analysis

In the landscape of traditional acoustic voice analysis, fundamental frequency (F0), sound pressure level (SPL), and parameters such as jitter, shimmer, and noise-to-harmonic ratio (NHR) have been pivotal [12]. F0 and SPL provide essential insights into pitch and loudness, forming the cornerstone of vocal assessment [3,13]. Conversely, jitter, which measures frequency perturbation, and shimmer, which quantifies amplitude perturbation, alongside NHR, a metric assessing the ratio of noise to harmonic components in the voice, delve into the finer details of voice signal stability, offering nuanced perspectives on the assessment of voice quality and voice disorders [3,12,13].

However, the reliability of these early parameters has been a subject of debate, primarily due to their susceptibility to significant variability, lack of robustness, and sensitivity to extrinsic factors such as frequency and intensity [14,15]. The inherent variability in voice production among individuals, coupled with the influence of recording conditions and analysis methodologies, can introduce a substantial inconsistency in measurements, as demonstrated by Maryn et al. [16]. This variability complicates the interpretation of jitter, shimmer, and NHR values, potentially confounding clinical assessments and research outcomes in different settings [17].

Moreover, the robustness of these microperturbation parameters is further challenged by their dependence on the underlying periodicity of the voice signal. In signals that exhibit a high degree of aperiodicity, common in severe dysphonia or substitution voices, the accuracy and relevance of jitter and shimmer measurements diminish significantly [3,18]. The sensitivity of these parameters to the fundamental frequency and intensity of the voice signal also raises concerns regarding their utility across a wide range of vocal intensities and pitches. Such sensitivity may lead to skewed assessments in cases where vocal parameters deviate from normative ranges, limiting the applicability of these measures in a diverse clinical population.

Another significant methodological limitation affecting jitter, shimmer, and NHR is that their calculation is predominantly based on sustained vowel sounds. This traditional approach encompasses a not negligible variability according to the vowel segment selection, introducing a certain risk of inconsistency across measurements and individuals [19]. Moreover, while methodically simpler, vowel analysis encounters limitations in ecological validity, as real-world voice use encompasses a dynamic range of vocal expressions beyond sustained vowels. The analysis of these parameters on sustained vowels, although informative, does not fully encapsulate the complexities of prosody [20]. On the other hand, in the context of continuous speech, the vocal signal presents a higher degree of variability and complexity, leading to potential inaccuracies in microperturbation measurements. This reduction in reliability stems from the parameters’ sensitivity to the periodicity of the voice signal, which is more pronounced in sustained vowels than in the variable pitch and intensity landscape of continuous speech.

In light of these limitations, the reliance on traditional parameters necessitates a cautious interpretation within the context of a comprehensive voice assessment. The recognition of these constraints has propelled the field toward the development and adoption of more robust, reliable, and multiparametric acoustic measures.

Cepstral measures for voice analysis

**Figure 1:**
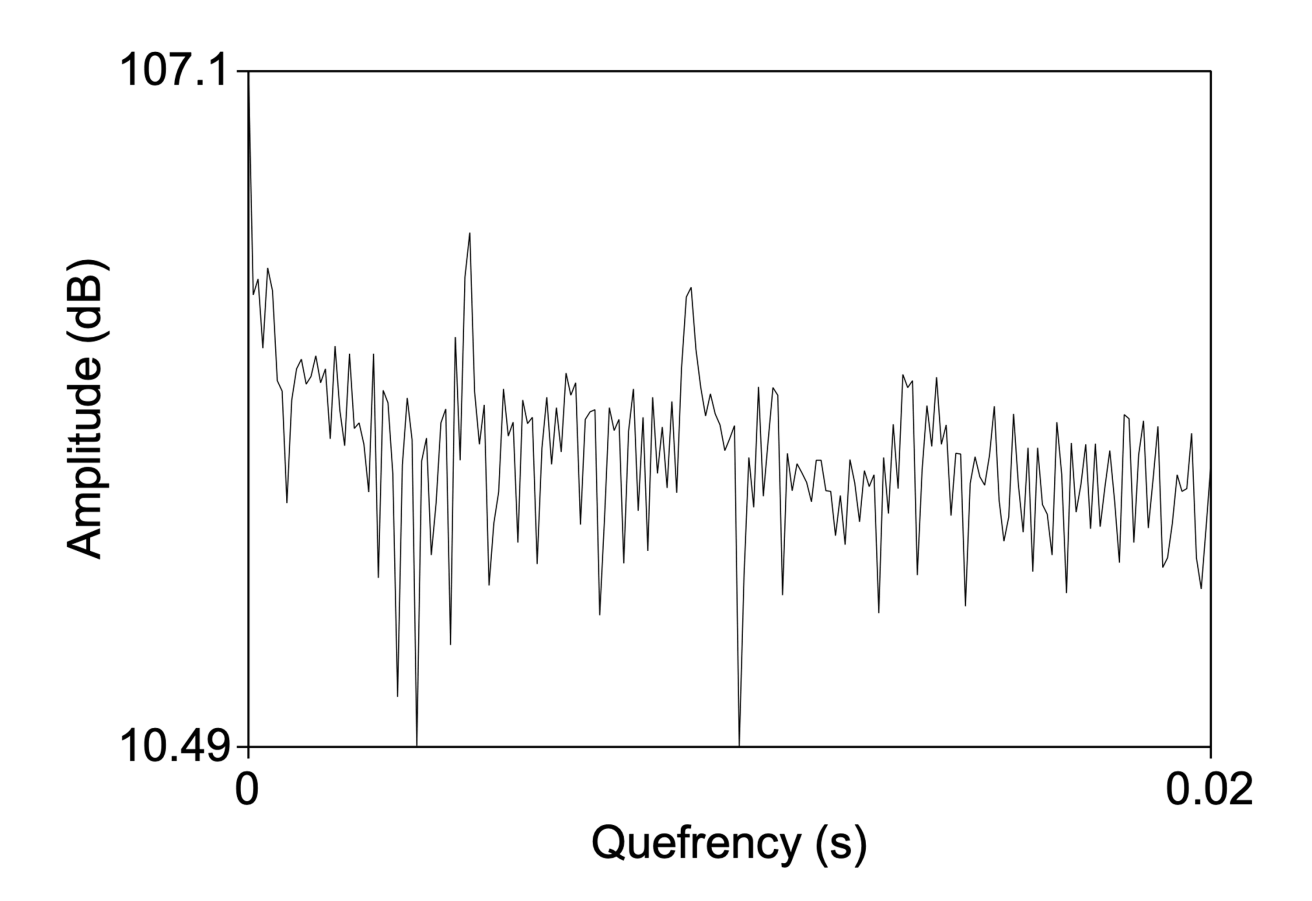
Graphic representation of a cepstrum. This figure was obtained using the free software PRAAT (www.fon.hum.uva.nl/praat/) by the author Marco Fantini.

Cepstral indices, particularly the cepstral peak prominence (CPP) and its smoothed variant, CPPs (smoothed cepstral peak prominence), represent a significant advancement in the acoustic analysis of voice [21]. These indices are derived from a mathematical representation of the voice signal known as the cepstrum, which is the result of taking the Fourier transform of the logarithm of the power spectrum of the voice signal [22], as shown in Figure 1. This process effectively captures and quantifies the periodicity of the vocal signal, offering insights into the harmonic structure and the presence of noise within the voice.

The CPP, and especially CPPs, have gained prominence due to their ability to provide a reliable, robust, and reproducible measure of voice quality, particularly in the assessment of dysphonia [23]. Unlike traditional parameters such as jitter and shimmer, cepstral measures do not require the voice signal to be perfectly periodic, making them more applicable across a wider range of voice qualities, including those with significant aperiodicity often observed in severely dysphonic voices [24,25]. Moreover, cepstral measures calculation is less susceptible to variations in recording conditions or the specific characteristics of the analyzed voice sample, leading to more consistent and reproducible results across different testing environments and populations [26].

The increasing utilization and trust in cepstral measures stem from their strong correlation with perceptual assessments of voice quality. Research has demonstrated that CPPs, in particular, is effective in distinguishing between normal and dysphonic voices both on sustained vowels and continuous speech, correlating well with the severity of vocal dysfunction [27]. This correlation is crucial in clinical settings where objective measures must align closely with perceptual evaluations to guide diagnosis and treatment. In summary, cepstral measures, particularly CPPs, have emerged in the last years as pivotal tools in the acoustic analysis of voice, providing a detailed and accurate assessment of voice quality and dysphonia [23,24].

Multiparametric constructs for voice analysis: a focus on the Acoustic Voice Quality Index

In the advancing field of voice analysis, the development of multiparametric constructs marks a significant stride toward a more holistic assessment of vocal quality. These constructs integrate various acoustic parameters to provide a comprehensive assessment of voice characteristics. Among the notable constructs are the Acoustic Voice Quality Index (AVQI) [28], the Cepstral Spectral Index of Dysphonia (CSID), and the Acoustic Breathiness Index (ABI) [29], which amalgamate multiple acoustic measures to evaluate specific aspects of voice quality, such as overall vocal quality and breathiness.

**Figure 2:**
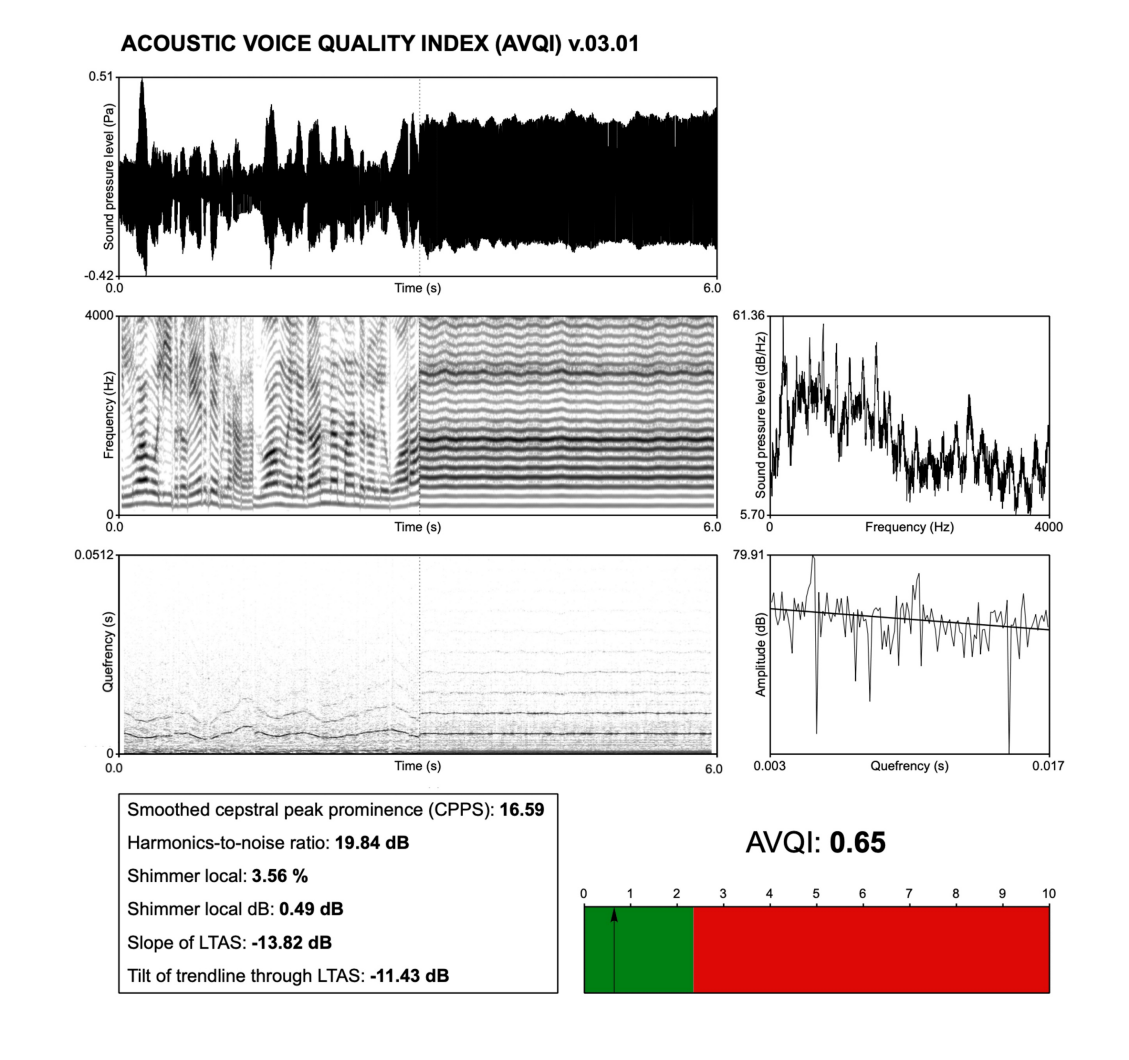
Example of the Acoustic Voice Quality Index (AVQI), version 03.01, with the Italian language cut-off 2.35. This figure was obtained using the free software PRAAT (www.fon.hum.uva.nl/praat/) by the author Marco Fantini.

A significant advancement in this realm is undoubtedly the AVQI, proposed by Maryn et al. [28]. The AVQI stands out as a robust, multivariable acoustic tool designed to quantify auditorily perceived overall voice quality by analyzing both sustained vowel sounds and voiced fragments of continuous speech [16]. It is calculated through a specific script run by the software PRAAT and is displayed as shown in Figure 2. Since its inception in 2010, AVQI has undergone refinements [30] and validation processes across several different languages worldwide, highlighting its universal applicability and reliability in voice quality assessment [31-39]. AVQI’s formulation includes a variety of acoustic measures, such as the HNR, shimmer, and CPPs, among others [28,30]. The AVQI emerges as a notable ecological measure of voice quality within the domain of acoustic analysis, principally due to its comprehensive approach that encompasses concatenated sustained vowels and continuous speech. This integration represents a significant advancement over traditional acoustic analyses, which predominantly focus on sustained vowels. The inclusion of continuous speech in AVQI’s assessment framework addresses a critical limitation of earlier methods, offering a more representative and holistic evaluation of vocal function as encountered in daily life.

The comprehensive validation and widespread adoption of AVQI were highlighted in a recent meta-analysis, which drew attention to its strong diagnostic accuracy and clinical utility [40]. The meta-analysis reported an area under the curve (AUC) of 0.937 and a Q* index of 0.874, demonstrating AVQI’s exceptional capability in diagnosing voice disorders. Furthermore, the analysis underscored AVQI’s concurrent validity and sensitivity to change, with weighted mean correlation coefficients of 0.838 and 0.796, respectively. These findings affirm the AVQI’s effectiveness in mirroring perceptual evaluations of voice quality and tracking changes over time, whether due to natural progression, surgical intervention, or therapy.

Current recommendations on acoustic voice analysis: where are we now?

Recent clinical recommendations on voice acoustic analysis are progressively advocating for the use of cepstral measures or multiparametric indices for voice analysis. Authoritative examples include the protocols proposed by the American Speech-Language-Hearing Association (ASHA) [8] and those by Behlau et al. in Brazil [41]. The first paper suggests CPP, the latter CPPs, AVQI, and CSID as acoustic voice quality parameters. These recommendations reflect a growing recognition of the limitations of traditional acoustic parameters and emphasize the superior reliability, robustness, and clinical utility of cepstral and multiparametric measures in assessing vocal function and disorders.

In contrast, the recent European guidelines, emerging from an expert consensus panel of the European Laryngological Society and Union of European Phoniatricians, continue to recommend the use of traditional measures such as jitter, shimmer, and NHR for voice analysis in clinical settings [42]. These guidelines notably lack mention of cepstral parameters and offer scant citations of multiparametric constructs despite mounting evidence supporting their efficacy in voice analysis. This discrepancy underscores a significant divergence in the adoption of advanced voice analysis techniques across different regions and expert groups. The persistent recommendation of traditional measures, which are increasingly recognized for their limitations in ecological validity and sensitivity, highlights the need for an updated consensus that integrates contemporary findings and methodologies.

The adoption of cepstral measures and multiparametric indices in voice analysis represents a significant advancement in our ability to accurately and comprehensively assess voice quality and voice disorders. These measures offer a more nuanced understanding of vocal function, facilitating effective diagnosis, monitoring, and treatment planning. Therefore, the integration of modern acoustic analysis techniques into international standards would not only enhance the accuracy and reliability of voice assessments but also promote consistency in voice care practices internationally, ultimately benefiting patients and clinicians alike.

Toward the future: artificial intelligence for voice analysis and voice restoration

The last decades witnessed an increasing recognition within the medical scientific community about the significance of artificial intelligence (AI) in healthcare. This growing awareness is driven by emerging evidence showcasing AI’s potential to revolutionize various aspects of health-related research and clinical practice. Deep learning (DL) is a specialized domain within AI and machine learning (ML). AI encompasses algorithms tailored for tasks that traditionally require human-like intelligence. A distinctive feature of DL is its capacity to autonomously identify significant features from training data, eliminating the need for pre-defined feature specifications. The foundation of most DL models is convolutional neural networks and recurrent neural networks, which draw methodological parallels to biological neural networks. These networks consist of multiple strata of interlinked neurons, where each neuron functions as a simple mathematical function, processing and weight-averaging the outputs from preceding layers. Data input is fed into the model, leading to the generation of the model’s prediction. This output is then compared against actual values, facilitating the computation of errors. The network adjusts its synaptic weights (connections between neurons) based on these errors. Connections pivotal to accurate predictions are strengthened, whereas less significant ones are assigned lower weights. The network ultimately calculates a probability for the given input. For instance, in a healthcare application, the input might be a patient’s voice recording, and the network might output a binary classification, such as distinguishing between a normal voice and a pathological voice [43].

DL has emerged as a transformative force across various healthcare domains, ranging from the analysis of electronic health records [44] to medical imaging [45], drug discovery [46], and multi-omics analysis [47], achieving accuracies comparable to, or surpassing, those of human experts. Considering voice analysis, DL is gaining traction in this field as well, with studies demonstrating its potential to revolutionize the screening and early diagnosis of voice pathologies and voice-related diseases (such as neurological disorders) [48], offering a non-invasive, efficient alternative to traditional diagnostic methods. A recent systematic review by Tessler et al. analyzed 14 studies on the application of AI in voice analysis, showcasing promising diagnostic accuracy. This highlights AI’s potential for early diagnosis and screening using vocal signals, including remote applications [43].

A step forward might be represented by the application of the so-called Transformer models to voice analysis [49]. Leveraging the self-attention mechanism, Transformers can discern patterns by comparing different pieces of data (tokens) even when widely spaced. This capability enables them to understand the context of each token effectively [50], making them suitable for analyzing voice data and capturing subtle nuances in speech patterns and intonations. Their parallel processing capability allows for the efficient handling of sequential information such as language. Initial studies [51,52] suggest that Transformers significantly improve voice-based diagnostics by identifying vocal features associated with specific pathologies. Furthermore, these models excel in handling diverse modalities simultaneously, promising a holistic understanding of an individual’s health status [53].

Apart from voice analysis and early diagnosis of voice-related pathologies, emerging applications of AI for voice restoration, particularly for patients with severely deteriorated voices due to conditions such as laryngectomies or cordectomies for laryngeal cancers [54] or neurological disorders [48], present intriguing possibilities. The impact of these conditions on voice quality significantly affects patients’ voice-related quality of life, especially in crucial areas such as telephone communication [55]. AI could play a key role in this context, fostering the development of systems aimed at real-time audio signal manipulation to restore clear and intelligible speech, both through smartphone applications [56] and wearable devices [57]. This advancement would greatly enhance communication abilities and quality of life for affected individuals.

## Conclusions

In the rapidly evolving scenario of voice acoustic analysis, there has been a significant progression from basic techniques, which were subject to high variability and limited reliability, to increasingly complex constructs and more robust methods. These advanced techniques are gradually overcoming the limitations inherent in wave perturbation measures, marking a notable evolution in the field. Moreover, the last years witnessed a clear trend toward the adoption of AI for voice analysis. This evolution signifies a potential shift from traditional methods to more advanced, AI-driven techniques. AI’s promise lies in its capacity for remote, efficient, and precise screening of voice-related pathologies, likely shaping the future of this field. This development could revolutionize the approach to voice health, offering new possibilities for screening, early detection, and intervention in vocal disorders.
